# Whether or not the weather matters: A retrospective review assessing the influence of weather on SARS-CoV-2 transmission

**DOI:** 10.1017/ash.2023.298

**Published:** 2023-09-29

**Authors:** Melissa Colaluca, Sean Harford, Ann Palmer, Ulysses Wu, Kaelin Wu, Matthew Kulowski

## Abstract

**Background:** Since the start of the COVID-19 pandemic, many variables have contributed to surges in cases such as the presence of variants, vaccination status, and comorbid medical conditions. However, other factors can be considered including temperature, precipitation, and periods in large congregations. The spike in SARS-CoV-2 infections during the winter has made it seem plausible that transmission may be affected by meteorological factors. A study by Birukov et al demonstrated that a 1°C increase in temperature was associated with a 3.08% reduction in daily new cases and a 1.19% decrease in daily new deaths. We propose that SARS-CoV-2 transmission will decline more rapidly when either precipitation or temperature is higher; thus, in warmer regions with less precipitation daily cases, hospitalizations and deaths will be lower. **Methods:** This is a retrospective study of statewide data in Hartford County, Connecticut, collected from May 2020 to June 2022 assessing percent positivity reported in daily case count, hospitalizations for COVID-19, and deaths from COVID-19 collected from the Connecticut Department of Public Health COVID-19 database. Information on weather conditions, including temperature and precipitation, were collected from the National Weather Service pertaining to Hartford County. Trends in variables related to patient outcomes were compared to weather conditions within the county of Hartford. Moreover, certain periods within the various seasons that typically involve large gatherings and public holidays (eg, New Year’s Day, Memorial Day, 4th of July, Labor Day, Thanksgiving, and Christmas Day) were further analyzed. **Results:** There appears to be an inverse correlation coefficient of −0.422, between confirmed daily cases and mean temperature in Hartford County, indicating that as temperature increases, confirmed cases decrease. This phenomenon is also observed with confirmed daily deaths and mean temperature, with a correlation coefficient of −0.463. Moreover, there is an even more significant relationship between hospitalization cases and mean temperature, with a correlation coefficient of −0.667. Furthermore, the year-end holidays (Christmas Day and New Year’s Day) were associated with a significant spike in confirmed daily cases, hospitalizations, and deaths.

However, the relationship between confirmed daily cases, hospitalized cases, and confirmed deaths against mean precipitation in Hartford County demonstrated no significant relationship, reporting correlation coefficients of −0.042, −0.044, and −0.044, respectively. **Conclusions:** Our available COVID-19 and weather data show that temperature is inversely correlated with daily cases, hospitalizations, and deaths. However, with regard to precipitation, there was no discernable relationship between the variables.

**Figure:**
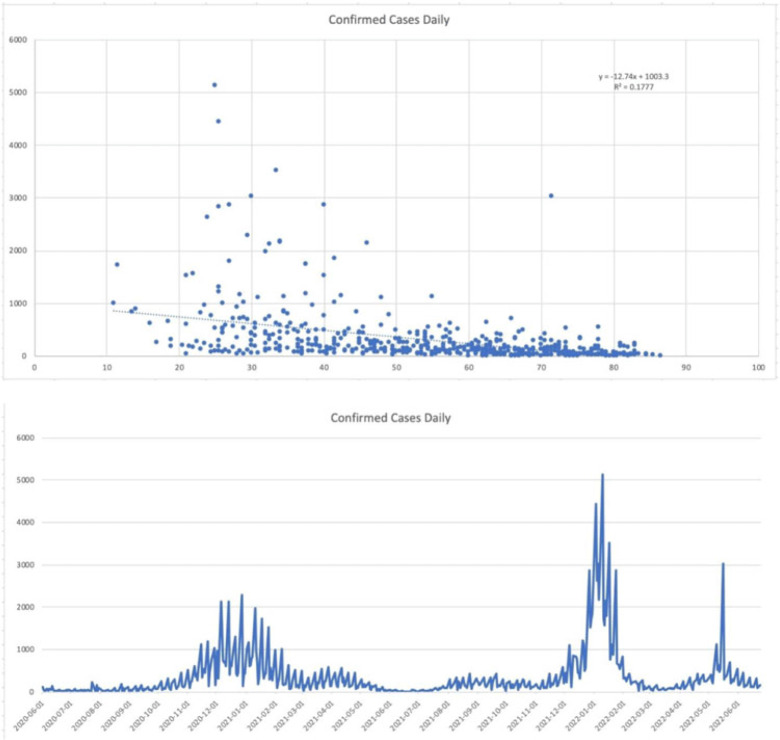

**Figure:**
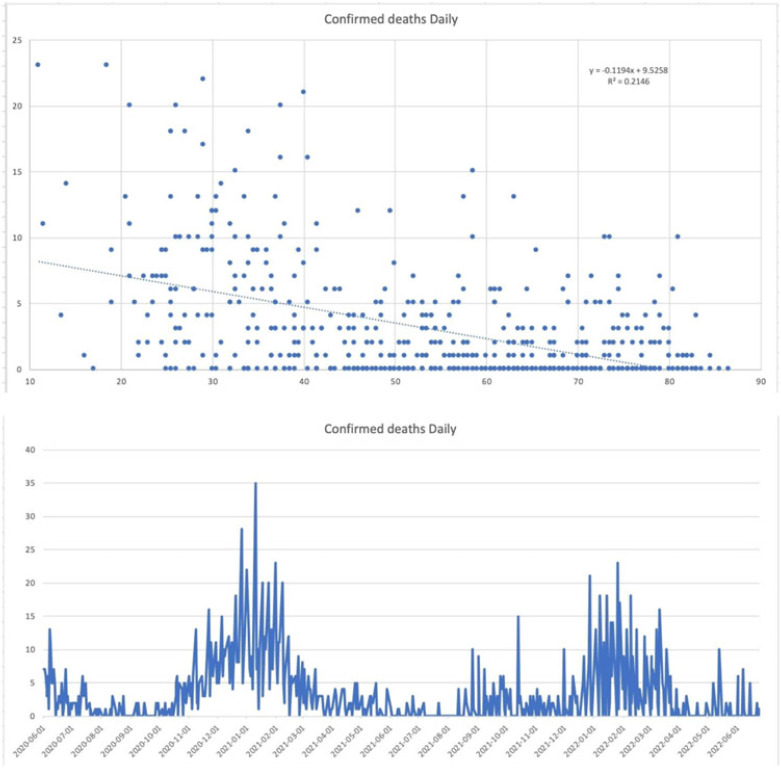

**Disclosures:** None

